# Development of a CT-Compatible, Anthropomorphic Skull and Brain Phantom for Neurosurgical Planning, Training, and Simulation

**DOI:** 10.3390/bioengineering9100537

**Published:** 2022-10-09

**Authors:** Marco Lai, Simon Skyrman, Flip Kor, Robert Homan, Victor Gabriel El-Hajj, Drazenko Babic, Erik Edström, Adrian Elmi-Terander, Benno H. W. Hendriks, Peter H. N. de With

**Affiliations:** 1 Philips Research, High Tech Campus 34, 5656 Eindhoven, The Netherlands; 2 Department of Engineering, Eindhoven University of Technology (TU/e), 5612 Eindhoven, The Netherlands; 3 Department of Clinical Neuroscience, Karolinska Institutet, 17177 Stockholm, Sweden; 4 Department of Neurosurgery, Karolinska University Hospital, 17164 Stockholm, Sweden; 5 Department of Biomechanical Engineering, Delft University of Technology, Mekelweg 2, 2628 CD Delft, The Netherlands; 6 Philips Healthcare, 5684 Best, The Netherlands

**Keywords:** anthropomorphic phantom, skull phantom, brain phantom, CT compatible phantom, neurosurgical simulation, endonasal skull-base surgery, brain biopsy, external ventricular drain

## Abstract

Background: Neurosurgical procedures are complex and require years of training and experience. Traditional training on human cadavers is expensive, requires facilities and planning, and raises ethical concerns. Therefore, the use of anthropomorphic phantoms could be an excellent substitute. The aim of the study was to design and develop a patient-specific 3D-skull and brain model with realistic CT-attenuation suitable for conventional and augmented reality (AR)-navigated neurosurgical simulations. Methods: The radiodensity of materials considered for the skull and brain phantoms were investigated using cone beam CT (CBCT) and compared to the radiodensities of the human skull and brain. The mechanical properties of the materials considered were tested in the laboratory and subsequently evaluated by clinically active neurosurgeons. Optimization of the phantom for the intended purposes was performed in a feedback cycle of tests and improvements. Results: The skull, including a complete representation of the nasal cavity and skull base, was 3D printed using polylactic acid with calcium carbonate. The brain was cast using a mixture of water and coolant, with 4 wt% polyvinyl alcohol and 0.1 wt% barium sulfate, in a mold obtained from segmentation of CBCT and T1 weighted MR images from a cadaver. The experiments revealed that the radiodensities of the skull and brain phantoms were 547 and 38 Hounsfield units (HU), as compared to real skull bone and brain tissues with values of around 1300 and 30 HU, respectively. As for the mechanical properties testing, the brain phantom exhibited a similar elasticity to real brain tissue. The phantom was subsequently evaluated by neurosurgeons in simulations of endonasal skull-base surgery, brain biopsies, and external ventricular drain (EVD) placement and found to fulfill the requirements of a surgical phantom. Conclusions: A realistic and CT-compatible anthropomorphic head phantom was designed and successfully used for simulated augmented reality-led neurosurgical procedures. The anatomic details of the skull base and brain were realistically reproduced. This phantom can easily be manufactured and used for surgical training at a low cost.

## 1. Introduction

Neurosurgical procedures rely on the surgeon’s operative skills, including a deep knowledge of the relevant anatomy. Various strategies are deployed to attain and develop the necessary surgical abilities and anatomical knowledge, including training on cadavers, in a VR environment, or on surgical phantoms. 

Traditionally, neurosurgical training has been performed on human cadavers. While this has the advantage of reproducing authentic anatomical conditions, it also requires certain facilities, is costly, and raises ethical and legal concerns [1]. 

In recent years, there has been a shift towards computer simulation-based training, such as virtual reality (VR) platforms combined with handheld devices [2]. Simulators can often measure performance and provide immediate feedback, but may lack in providing realistic haptic feedback, and life-like 3D visualization. Moreover, they often do not allow the use of actual surgical instruments and come at a substantial cost [3]. 

The use of anthropomorphic phantoms could solve many issues related to cadaveric or VR models. Pre-operative CT or MRI images of the individual patient may be used to create a phantom that accurately replicates the patient-specific anatomy. Moreover, realistic anthropomorphic and CT-compatible phantoms can be used for initial testing of new technologies, such as navigation systems, before costlier cadaveric studies are conducted. The increased availability and quality of 3D printing has paved the way for complex anatomical models with the required accuracy and tactile properties. The most common phantoms are 3D-printed skulls in different plastic materials [4,5,6,7]. Phantoms have also been created for the modelling of vascular structures, including cerebral artery aneurysms [8] and complex anatomical variations of the internal carotid artery [9]. In ear, nose, and throat (ENT) surgery, 3D-printed phantoms have been used for testing AR systems in middle ear surgery [10] and for simulation of sinus and nasal cavity surgery, where the nasal cavities were included in the phantoms [11]. To simulate brain biopsies, brain phantoms have been built to mimic brain tissue with target lesions placed inside a cadaveric human skull [12]. An anatomically and mechanically realistic brain phantom was proposed and constructed by Chen and Shih, using polyvinyl alcohol cryogel (PVAC), a material widely used in the production of medical imaging phantoms for its mechanical similarities to soft tissues [13].

Even though several examples of anthropomorphic phantoms exist, there is no commercially available phantom that produces realistic results when imaged with CT and meets the requirements for the simulation of neurosurgical procedures, including navigated ones. The aim of this study was to design an anatomically accurate anthropomorphic skull and brain phantom optimized for CT for the planning, training, and simulation of neurosurgical procedures, with a specific focus on endonasal skull-base surgery, needle biopsies, and EVD placement [14].

## 2. Materials and Methods

### 2.1. Phantom Requirements for Realistic Surgical Simulations

Endonasal skull-base surgery offers a minimally invasive approach to remove tumors of the skull base, of which pituitary tumors are the most common type. In clinical practice, surgery is performed through the nasal cavity, using rigid instruments and a rigid endoscope to gain access to and visualize the surgical field. To design a phantom for the realistic simulation of endonasal skull-base surgery, several anatomical structures need to be present. The phantom must contain a correct representation of the nasal cavity including nasal turbinates, nasal meatuses, the nasal septum, sphenoidal sinus, natural sphenoidal ostia, sphenoid inter-sinus septum, and sella turcica, as well as critical neurovascular structures such as the optic nerves, optic chiasm, pituitary gland, and carotid arteries. For training of tumor resection, a realistic model of a tumor and the characteristic anatomical changes associated with tumor growth, must also be represented.

Brain biopsies are performed to obtain tissue samples necessary to diagnose tumors and other pathologies when surgical resection is not feasible. A small hole is drilled through the skull, after which a biopsy needle is introduced into the brain and samples for tissue diagnosis are collected. The needle is usually navigated based on preoperative CT or MRI. A combined skull and brain phantom would be needed to simulate brain biopsies. The brain phantom should have anatomical, mechanical, and radiological properties closely replicating those of a real brain.

### 2.2. Computational Model

A computational model of the skull and brain phantom was created using cone beam computed tomography (CBCT) and a T1-weighted MRI scan of a cadaver. The images were acquired at the Cincinnati Children’s Hospital Medical Center, Ohio, United States. Informed consent for body donation for research and educational purposes had been given by the donors, and the study was performed in adherence with all ethical guidelines for human cadaver studies.

**Figure 1:**
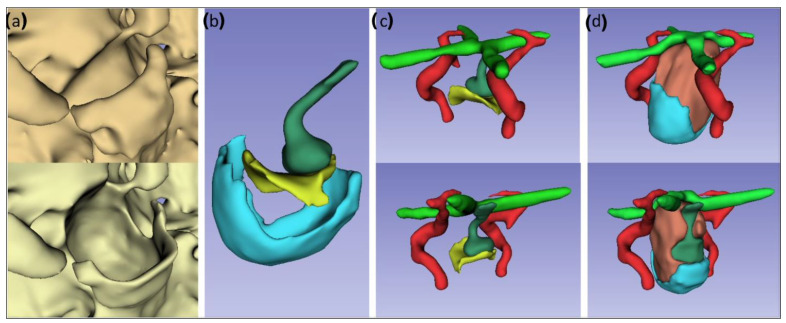
Modifications of the computational model of the cadaveric model. (**a**) Comparison between the original anatomy (top) and the pathological shape of the skull base (bottom). (**b**) Comparison of the original (yellow) and enlarged (turquoise) floor of the sella turcica. The original anatomy of the pituitary gland is presented in dark green. (**c**) Anterior and posterior view of the original anatomy of the sella turcica (yellow), internal carotid arteries (red), optic chiasm (light green), and pituitary gland (dark green). (**d**) Anterior and posterior view of the modified computational model of a pathological condition resulting from a pituitary tumor (coral) with an enlarged sella turcica (turquoise), a compressed pituitary gland (dark green), and a superiorly dislocated optic chiasm (light green).

**Figure 2:**
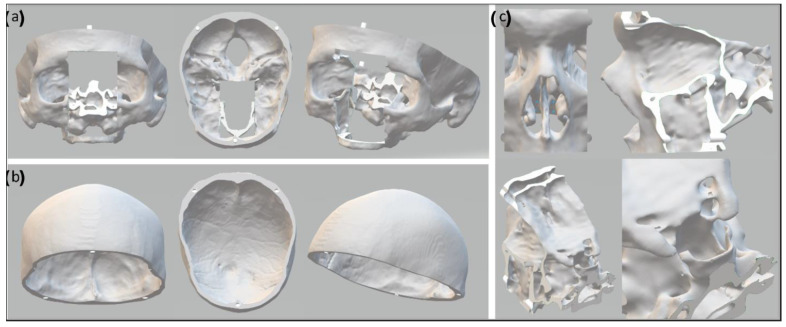
Computational model of the skull segmented from the CBCT image. The computation model of the skull (**a**) is modified to allow the removal of the top (**b**) for placement of the brain. The nasal cavity (**c**) is modular and can be replaced after surgical simulation.

**Figure 3:**
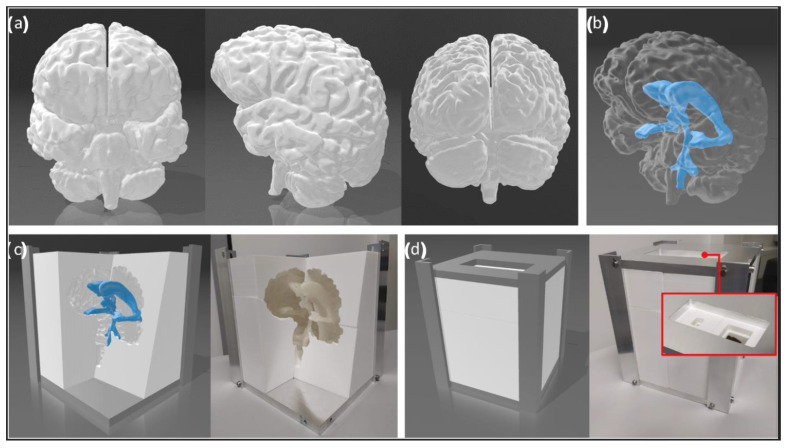
(**a**) Computational model of the brain, segmented from the MR image. (**b**) Computational model of the ventricles, located inside the brain. (**c**) Inside view of the computational model of the brain mold and corresponding inside view of the 3D-printed brain mold. (**d**) Outside view of the computational model of the brain mold and corresponding outside of the 3D-printed brain mold. The red frame shows the large and small openings at the top.

The CBCT and MR images were co-registered, and the open-source computer software 3D Slicer [15] (http://www.slicer.org, (accessed on: 30 January 2021)) was used for segmentation. The computer-generated model was evaluated and adjusted to verify the presence of all the required anatomic features. The CBCT image was used to model the bony structures, including the nasal cavity. The brain, the pituitary gland, optic chiasm, and carotid arteries were segmented from the T1-weighted MRI scan (Figure 1), after which the segmented structures were inverted to negative images to create molds for the phantom’s structures (Figure 2 and Figure 3).

The anatomical structures segmented from the cadavers were modified to simulate the presence of a pituitary tumor. A pituitary tumor originates from cells of the pituitary gland and results in a growing mass that affects the surrounding tissues. As the tumor grows, the sella turcica, i.e., the bony structure in which the pituitary gland is seated, is enlarged and the surrounding bone thinned out. The normal pituitary gland is compressed, and the optic nerves and optic chiasm are dislocated upwards (Figure 1).

### 2.3. The Skull Phantom

The skull phantom was printed using an Ultimaker 3 3D printer (Ultimater BV, Utrecht, Netherlands). To avoid having to reprint the whole phantom for every endonasal surgery simulation, the nasal cavity and frontal skull base were designed and printed as an exchangeable module. Thus, after a simulated surgical procedure, the modular part could be replaced with a new one. The upper part of the calvarium was designed to be removable to allow the placement and exchange of the brain phantom inside the skull phantom (Figure 2). Water-soluble polyvinyl alcohol (PVA) was used as a printing support material, and the printed parts were left overnight to let the PVA dissolve, after which they were polished with sandpaper to remove any imperfections.

Three low-cost materials were considered for building the skull phantom: gypsum powder, PLA (polylactic acid), and PLA+CaCO_3_ (calcium carbonate). Gypsum powder has a comparable radiodensity to bone, with Hounsfield unit (HU) values ranging from 1000 to 3000 [16]. PLA is a standard material used for FDM (fused deposition modeling), with properties ideal for 3D printing, but with a radiodensity around 0 HU. By mixing PLA with CaCO_3_, the HU of the print was increased to approximate the CT attenuation of bone. A sample of each material was scanned with CBCT (Allura Clarity Flexmove, Philips Healthcare, Best, The Netherlands) and compared to real bone tissue attenuation.

### 2.4. The Brain Phantom

The brain phantom was cast in a 3D-printed PLA mold that was divided into 8 segments held by a support structure of aluminum, to facilitate the ejection of the cast after molding. An opening at the top of the mold was used to pour in the liquid material, and another opening allowed the simultaneous release of air (Figure 3). 

The brain phantom was created using a mixture of water, coolant, polyvinyl alcohol (PVA, Sekisui SELVOL™ Polyvinyl Alcohol 165), and barium sulfate (BaSO_4_, SIGMA-ALDRICH^®®^), using one freeze-thaw cycle at −25 °C. The PVA mixture was prepared as previously described by Chen and Shih [13]. The coolant prevented the 3D-printed mold from breaking due to expansion of the PVA mixture during freezing. 

To mimic the CT attenuation of brain tissue, barium sulfate was added to the PVA mixture. A dilution series of barium sulfate was performed to evaluate radiodensity: barium sulfate was added to reach 0, 0.1, 0.2, 0.3, 0.4, and 0.5% by weight. The radiodensities of mixtures with different percentages of barium sulfate were tested with CBCT and compared to the attenuation of white and gray matter of real brain tissue, respectively [17].

The stiffness of the brain phantom material could be controlled by repeated freeze-thaw cycles or adjustment of the concentration of PVA. The mechanical properties of several PVA mixtures were tested to mimic brain tissue elasticity. The tested mixtures contained 3%, 4%, 5%, and 6% PVA, the rest was water and coolant in a proportion of 60% water and 40% coolant. The PVA samples had a thickness of 20–25 mm and were compressed uniaxially by a linear stage, which exerted its force via a round object with a diameter of 30 mm (contact area 706.85 mm^2^). All tests were carried out at room temperature. The stress-strain curve and elastic modulus of each mixture were measured and compared with the stress-strain response of porcine brain tissue at a strain-rate of 0.01/s as described by Li et al. [18], and with the stress-strain response of human brain tissue at a strain-rate of 0.083/s as described by Forte et al. [19].

### 2.5. The Skull-Base Structures

**Figure 4:**
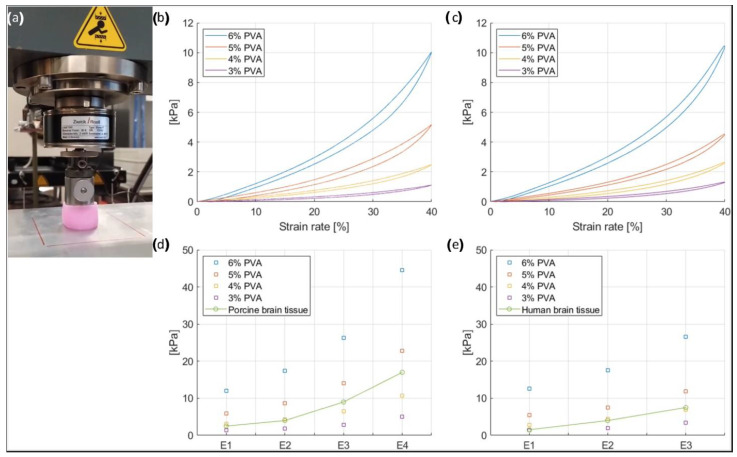
(**a**) PVA sample under test. (**b**) Strain-rate curves of the mechanical tests on the PVA samples at 0.01/s. (**c**) Strain-rate curves of the mechanical tests on PVA samples at 0.083/s. (**d**) Elastic moduli (Ei) of PVA samples at 0.01/s, compared to porcine brain tissue properties found in the literature. (**e**) Elastic moduli (Ei) of PVA samples at 0.083/s, compared to human brain tissue properties found in the literature.

The models of the pituitary gland, pituitary tumor, optic nerves, optic chiasm, and internal carotids were cast in 3D-printed resin molds that were designed in two separate pieces, in order to facilitate the extraction of the casting. (Figure 4). The resin material was chosen to allow a high printing accuracy (0.05 mm), since the structures were small (5–30 mm). The PVA material was injected inside the mold using a syringe. Several holes were added to the molds to allow air to escape during casting. Structures were colored for realism, by adding small amounts of colored inks to the mixtures.

### 2.6. Validation Step

The phantom described in this study was evaluated by five neurosurgeons with 10 to 20 years of clinical experience—the same neurosurgeons were also consulted during the design and manufacturing process, allowing improvements through an iterative approach of tests, evaluations, and adjustments. All participating neurosurgeons were from the same department and had previous experience with surgical simulations on phantoms and cadavers. Multiple procedures, including endonasal skull-base surgery, brain biopsies, and EVD placement were performed. 

Each neurosurgeon evaluated the phantom individually and independently. No standardized evaluation tools were used. Instead, a qualitative approach was used emphasizing the participating surgeons’ experience, and mainly addressing the following points: (i) the realism of the skull, brain, and skull-base structures, in terms of both appearance and texture, (ii) the durability of the materials used in the context of surgical manipulation and simulation including drilling, (iii) the ease with which the modular components could be replaced, and (iv) the overall usefulness of the phantom. The final model was used in a systematic evaluation of navigated biopsies using a novel system based on intraoperative 3D imaging.

## 3. Results

### 3.1. Mechanical Properties of the Brain Phantom

Figure 4a depicts a PVA sample under test. In Figure 4b,c, the stress-strain loop responses of the brain phantom samples to compression and decompression cycles are shown at 0.01/s and 0.083/s, respectively. The samples contained PVA concentrations of 3, 4, 5, and 6% PVA respectively. The strain-rate hysteresis loops showed that after compression, some energy is dissipated internally as friction (heat) during the unloading phase. To allow a comparison with the mechanical properties of the porcine brain as described by Li et al. [18] and the human brain as described Forte et al. [19], the elastic modulus (E) was calculated (Figure 4d,e). The elastic modulus is equal to the slope of the stress-strain curve and was calculated for four intervals of the stress-strain curve at 0.01/s and 0.083/s; E1 was defined as the elastic modulus for strain rates of 0–10%, E2 for strains of 10–20%, E3 for strain rates of 20–30%, and E4 for strain rates of 30–40%. Since E4 was not provided for the human brain tissue, the comparison is made between E1, E2, and E3. Additionally, the strain-rate curve at 0.01/s showed that at lower strains, represented by the elastic moduli E1 and E2, the 4% PVA mixture had a response very similar to the porcine brain, as represented by the overlap of the yellow and green boxes on the graph (Figure 4d). In addition, as indicated by the graph, at higher strains—E3 and E4—the sample was stiffer. In contrast, the strain-rate curve at 0.083/s showed that at lower strains, represented by the elastic modulus E1, the 3% PVA mixture had a response very similar to the human brain, as represented by the overlap of the purple and green boxes on the graph (Figure 4e), while at higher strains—E3 and E4—the 4% PVA mixture sample was more similar to the human brain tissue. Since the mixture with 4% PVA showed results comparable to the porcine brain and human brain tissue, it was chosen for the manufacturing of the brain phantom. Therefore, the final composition of the mixture is 4% PVA and 96% water and coolant, of which the latter are in proportions of 60% and 40%, respectively.

### 3.2. Radiodensity of the Skull and Brain Phantom

#### 3.2.1. Radiodensity of the Skull Phantom

**Figure 5:**
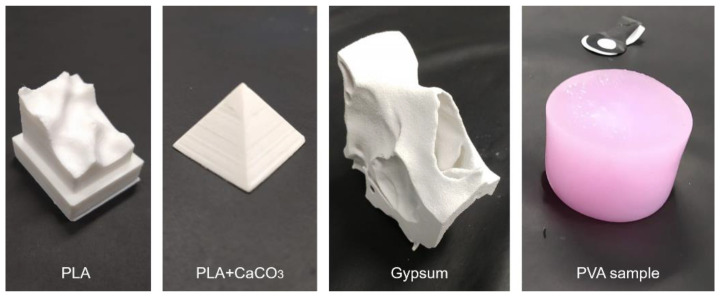
Pictures of the X-ray sample materials that have been tested.

**Figure 6:**
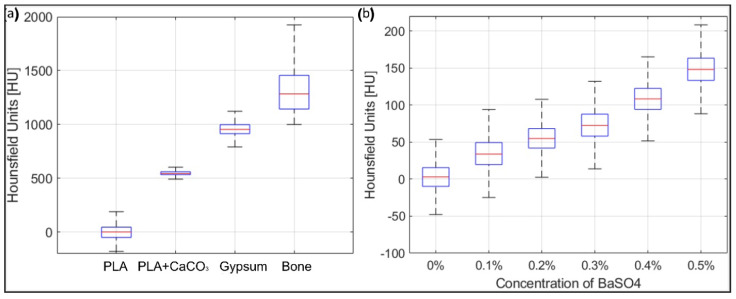
(**a**) Boxplots related to the X-ray attenuation, in Hounsfield units, of the materials for building the skull phantom. (**b**) Boxplots related to the X-ray attenuation, in Hounsfield units, of the mixtures for producing the brain.

Figure 5 shows the pictures of the X-ray sample materials that have been tested. The radiodensity measurements of the materials considered for the skull phantom, as well as real bone (skull cadaver) are presented in Figure 6a. PLA had a median value of 3 HU, PLA+CaCO_3_ of 547 HU, and gypsum powder of 955 HU. When choosing materials for the skull phantom, the ease of production, cost, and durability for repeated usage were also considered. The gypsum powder exhibited a radiodensity closer to real bone, but the gypsum 3D prints were too fragile for surgical simulations. Instead, the PLA+CaCO_3_ mixture was chosen since it fulfilled both the practical requirements and showed a radiodensity comparable to bone.

#### 3.2.2. Radiodensity of the Brain Phantom

After testing the mechanical properties of the brain phantom, the experiments on the radiodensity of the brain phantom are carried out with a mixture containing 4% PVA. The median CT attenuation of the human brain is approximately 29 HU for white matter and 35 HU for gray matter [17]. Figure 6b shows the radiodensity of the six samples. The PVA and water mixture had a radiodensity of 3 HU. With the addition of barium sulfate, the CT attenuation gradually increased from 33 HU (0.1% BaSO_4_) to 148 HU (0.5% BaSO_4_). The radiodensity of the 0.1% BaSO_4_ mixture was slightly lower than that of the gray matter (35 HU) and was chosen for the brain phantom. 

### 3.3. The Final Skull and Brain Phantom

**Figure 7:**
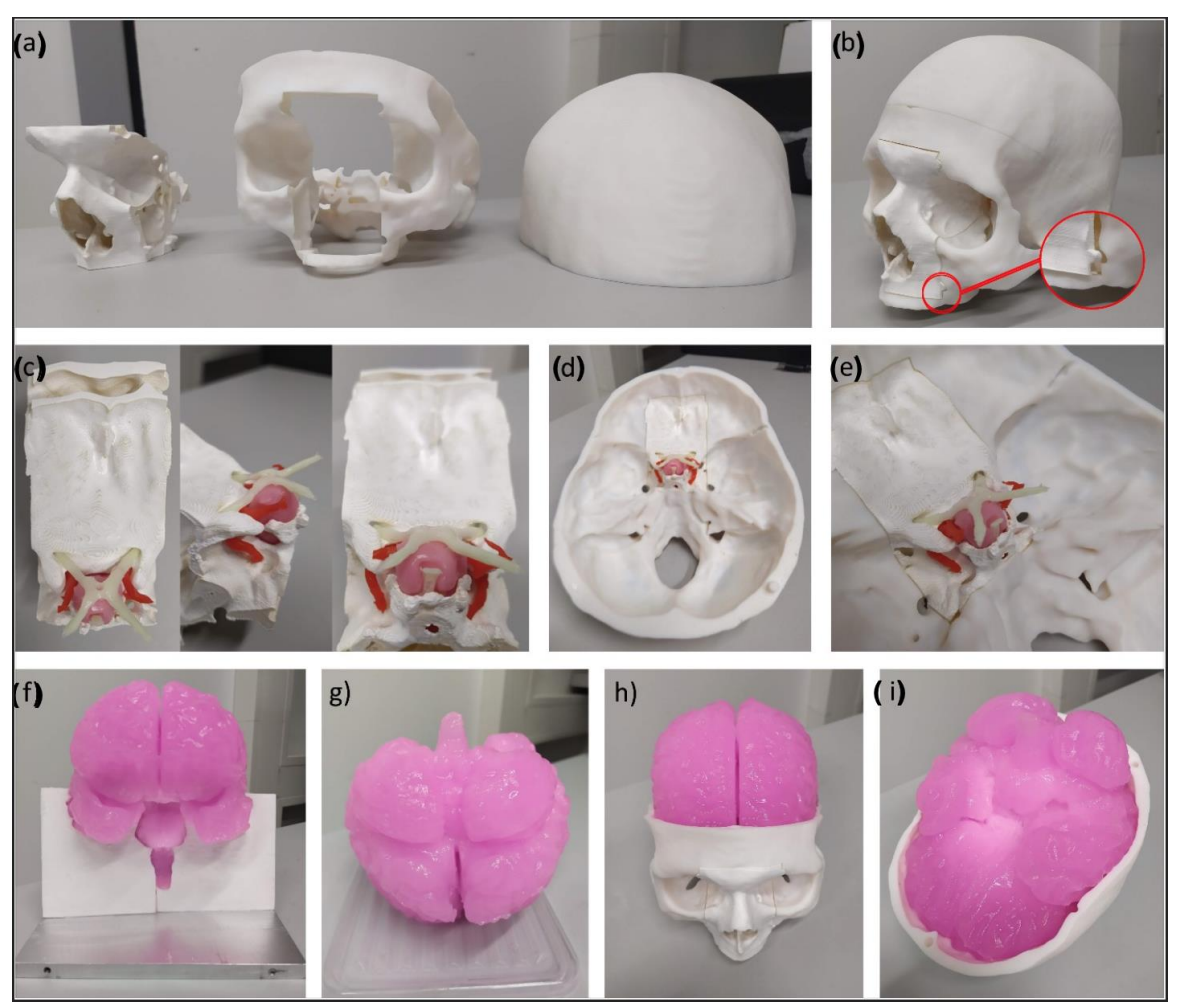
Final head phantom. (**a**) Skull phantom disassembled into its three parts. (**b**) Assembled skull and zoom-in view of the side groove of the maxilla that improves the fit of the nasal cavity insert into the skull phantom. (**c**) Upper and side views of the nasal cavity and skull-base inserts. (**d**) Back view of the nasal cavity inserted inside the skull. (**e**) Zoom-in of the nasal cavity inserted inside the skull. (**f**) Front view of the brain phantom, partially inside the mold. (**g**) Back view of the brain phantom. (**h**) Brain phantom placed inside the skull. (**i**) Brain phantom viewed from below, inserted inside the calvarium of the skull.

Figure 7 shows the 3D-printed head phantom with the skull, brain, and anatomical structures of the skull base. The skull can be disassembled into three pieces: (i) the bottom part including the facial skeleton, skull base, and bottom of the calvarium; (ii) the top part consisting of the upper part of the calvarium; and (iii) the replaceable nasal cavity with the medial frontal skull base (Figure 7a). The design allows the placement and replacement of the brain phantom inside the skull, as well as the nasal cavity, which fits well inside the phantom and stays in position without moving, with the help of grooves on the sides (Figure 7b). Tumor, arteries, nerves, and the pituitary gland are placed at the base of the skull, on top of the nasal cavity component (Figure 7c–e). The brain fits inside the skull and the anatomical features are well delineated, including the brainstem and the cerebral hemispheres, with gyri and sulci present on its entire surface (Figure 7f–i). 

The final prototype was assessed by experienced neurosurgeons, who found the head phantom to meet the expectations of both anatomical and surgical realism. A detailed quantitative assessment of the model with respect to the simulated surgical outcomes has been reported in a previous study [20].

### 3.4. Validation 

**Figure 8:**
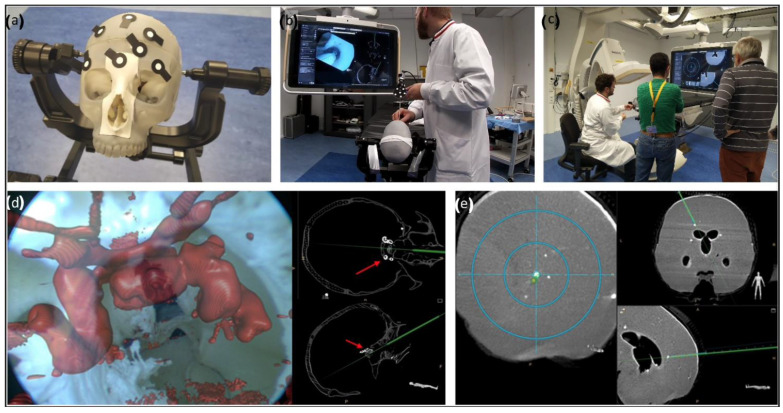
(**a**) Head phantom blocked on a clamp, equipped with optical markers. (**b**) Augmented reality-navigated endonasal skull-base surgery simulation. (**c**) Augmented reality-navigated brain biopsy simulations. (**d**) Skull-base surgery simulation. The endoscopic view is augmented with the critical structures of interest, as well as the trajectory of the endoscope being overlaid onto the CBCT image. (**e**) Brain biopsy simulation. A tracked needle is inserted inside the brain, throughout the skull, and its trajectory is overlaid onto the CBCT image.

The validation of the phantom was performed by five neurosurgeons, through navigated simulations of endonasal skull-base surgery, brain biopsies, and EVD placements (Figure 8). The final evaluation of the phantom concluded that the anthropomorphic skull and brain phantom satisfied the basic requirements for a life-like simulation model and provided an accurate CT attenuation ideal for simulations using surgical navigation based on 3D imaging.

## 4. Discussion

In this study, an anthropomorphic CT-compatible skull and brain phantom was developed. This phantom provides an accurate representation of critical neurovascular structures at the skull base and a removable nasal cavity. To achieve authenticity and maintain low costs, the materials used for production were carefully selected, and tested in different concentrations and compositions to match criteria formulated from published data. In addition, the model was tested and evaluated by neurosurgeons for representativity and utility [20].

The materials that were selected to replicate the skull bone included PLA, a mixture of PLA and CaCO_3_, and gypsum powder. Even though testing revealed that the median gypsum powder radiodensity was the closest to that of real bone, the gypsum prints were too fragile for surgical manipulation. Instead, the combination of PLA and CaCO_3_ was found to be a good compromise between authenticity, ease of production, cost, and durability.

For the brain phantom, imaging experiments on the PVA samples revealed that a small amount of BaSO_4_ (0.1%) was sufficient to achieve CT attenuations similar to that of brain tissue. The evaluation of the mechanical properties of the different PVA mixtures showed that the 4% PVA mixture had a strain response similar to that of porcine brain tissue, especially at low strains.

The anthropomorphic cranium made of PLA+CaCO_3_ was combined with the brain made of 4% PVA with 0.1% BaSO_4_ and subsequently evaluated by a group of neurosurgeons. They found the phantom had sufficient realism and anatomical detail to allow the simulation of neurosurgical procedures. They indicated the usefulness of such a phantom in the preparation of patient-tailored surgical approaches for complex cases. Arguably, a patient-specific phantom could provide an advantage compared to 2D- or 3D-image-based surgical planning, by simplifying the understanding of patient positioning and limitations, while allowing for the visualization of different approaches [21]. This may be especially valuable in cases of abnormal anatomy, such as that seen after previous surgery or tumor growth.

For neurosurgical training, the head phantom could be part of a realistic educational environment for endoscopic endonasal skull-base surgeries, brain biopsies, or EVD placements. To assist in relaying feedback to trainees, the phantom provides the possibility of confirming the accuracy of biopsies or drain placements using standard X-ray technology. The neurosurgical discipline is steadily evolving, and novel technologies push the boundaries of what is possible. In this context, senior surgeons may benefit from an anthropomorphic model to evaluate and practice new approaches, methods, and technologies. Moreover, the phantom could easily be modified to become better suited for the evaluation of a desired procedure such as the excision of intra- or extra-axial brain lesions [22,23] or the treatment of complex cerebrovascular cases [24].

Another use of this phantom is within the field of research and product development [25,26]. The phantom was originally intended for simulation in research settings. In the earlier steps of the development of new surgical equipment, testing and experimenting can be performed on models, postponing or averting the need for costly trials using cadaveric models. By virtue of its realistic imaging properties, the phantom can be used in the development and testing of navigation solutions that require imaging-based set-ups. In fact, the phantom has recently been used to test the accuracy and efficacy of an augmented reality-based surgical navigation system for brain biopsies or EVD placement [20]. This use of the phantom provides a safe and cost-effective approach to evaluate experimental tools and solutions [27].

Several neurosurgical 3D training models have previously been described [28,29]. The ones that most closely resemble the current phantom were described by Grillo et al. [30] and Craven et al. [31]. Although they were able to create a patient-specific 3D skull and brain phantom that was highly rated among health care professionals including neurosurgeons, neither Grillo et al. nor Craven et al. included a representation of the skull base, nasal cavity, pituitary, or adjacent neurovascular structures, making the simulation of skull-base and endonasal procedures impossible [30,31]. Although the mechanical properties of these phantoms were assessed for resemblance to real tissue [30], they did not account for radiodensity metrics when choosing the products used for manufacturing [27]. However, a latex balloon was added to cover the brain phantom and mimic the dura, a feature that was not included in the phantom presented in this study [30]. Additionally, another study proposing a similar model also featured a dura covering the 3D brain to enhance the surgical simulation experience [32]. Other synthetic head models lack an accompanying brain phantom [33,34,35,36,37,38], or cannot be used to perform simulation surgeries [33,34,39].

As seen, only a handful of models similar to ours have been described in the literature. Even among the current studies, information regarding cost was often lacking. Only one study, by Waran et al., reported the manufacturing cost of their model [32]. Although describing a similar concept as presented here, their prototype lacked both endonasal and skull-base modules. Waran et al. estimated the final cost of the product at 2600 USD, counting 2000 USD for the reusable skull and 600 USD for the disposable brain [32]. In our study, the skull and skull base cost 215 USD each, while the exchangeable nasal cavity cost 130 USD. In total, the cranium cost 560 USD. The materials used in the manufacturing of the brain and skull-base structures only cost around 10 USD, bringing the total cost of this phantom to about 570 USD. However, the final price tag does not include any indirect costs associated with the manufacturing of the product, such as molds, equipment, and labor. In fact, we estimate the cost of the molds utilized to reach up to 2130 USD in price. However, larger scale or mass production may certainly aid in reducing cost margins, as the molds, for example, may be reused, hence making the product even more affordable.

In conclusion, the anthropomorphic skull and brain phantom described in this study presents the features of anatomical, radiological, and surgical realism, combined with cost-efficiency and durability. One of the key advantages of this phantom is that it is based on a computer model, which makes it easily customizable to serve different cases and applications. In addition, the durability of the chosen materials, and the removable and replaceable pieces, allow for repeated simulations without the need to rerun the whole process of casting and printing after each simulation. The phantom lends itself to the simulation of a diversity of procedures on the skull, skull base, and brain for the teaching and training of surgeons, and the development of surgical tools and solutions [20]. Finally, the phantom may also prove useful in the area of patient education, where its use is currently being tested [40,41].

### Limitations

An inherent limitation to the in-house manufacturing of prototypes lies in the process of segmenting the details of the phantom, as well as the actual 3D printing of the model, which are both time-consuming. The use of advanced, artificial intelligence-powered automatic or semi-automatic methods for image segmentation could possibly improve and speed up this process, enabling the more routine use of this phantom [42].

## 5. Conclusions

In neurosurgery, the value of synthetic models has recently been acknowledged, although such models are frequently denounced as lacking adequate realism when compared to other simulation techniques [29]. In this study, we aimed to challenge these claims by designing a realistic and CT-optimized anthropomorphic head phantom. The phantom was created using authentic human CBCT and MR images. The skull, the neurovascular structures at the skull base, and the brain parenchyma were also realistically recreated. A series of experiments pertaining to the mechanical and radiological properties of the materials used were performed to achieve a realistic consistency and an accurate radiodensity of the materials. Both durability and cost were taken into consideration. Subsequent validation experiments during the present and previous studies [20] have shown the utility of this model in training for a variety of procedures such as brain needle biopsy, EVD placement, skull-base surgery, and also including all sorts of augmented reality-navigated neurosurgical procedures.

## Data Availability

The datasets supporting the conclusions of this article may be provided upon reasonable request.

## Previous presentation

An abstract based on this manuscript was submitted to the International Society for Optics and Photonics, SPIE, Congress of Medical Imaging 2021: Marco Lai, Simon Skyrman, Flip Kor, Robert Homan, Drazenko Babic, Erik Edström, Oscar Persson, Gustav Burström, Adrian Elmi-Terander, Benno H. W. Hendriks, Peter H. N. de With, “Development of a CT-compatible anthropomorphic skull phantom for surgical planning, training, and simulation”, SPIE medical imaging, Volume 11601, 2021.

## References

[1] Breimer G.E., Bodani V., Looi T., Drake J.M. (2015). Design and evaluation of a new synthetic brain simulator for endoscopic third ventriculostomy. J. Neurosurg. Pediatr..

[2] Iop A., El-Hajj V.G., Gharios M., de Giorgio A., Monetti F.M., Edström E., Elmi-Terander A., Romero M. (2022). Extended Reality in Neurosurgical Education: A Systematic Review. Sensors.

[3] Lange T., Indelicato D.J., Rosen J.M. (2000). Virtual Reality in Surgical Training. Surg. Oncol. Clin. N. Am..

[4] Mirota D.J., Uneri A., Schafer S., Nithiananthan S., Reh D.D., Ishii M., Gallia G.L., Taylor R.H., Hager G.D., Siewerdsen J.H. (2013). Evaluation of a System for High-Accuracy 3D Image-Based Registration of Endoscopic Video to C-Arm Cone-Beam CT for Image-Guided Skull Base Surgery. IEEE Trans. Med. Imaging.

[5] Gibby J., Cvetko S., Javan R., Parr R., Gibby W. (2020). Use of augmented reality for image-guided spine procedures. Eur. Spine J..

[6] Chu Y., Yang J., Ma S., Ai D., Li W., Song H., Li L., Chen D., Chen L., Wang Y. (2017). Registration and fusion quantification of augmented reality based nasal endoscopic surgery. Med. Image Anal..

[7] Bong J.H., Song H.-J., Oh Y., Park N., Kim H., Park S. (2018). Endoscopic navigation system with extended field of view using augmented reality technology. Int. J. Med. Robot. Comput. Assist. Surg..

[8] Nagassa R.G., McMenamin P.G., Adams J.W., Quayle M.R., Rosenfeld J.V. (2019). Advanced 3D printed model of middle cerebral artery aneurysms for neurosurgery simulation. 3D Print. Med..

[9] Govsa F., Yagdi T., Ozer M.A., Eraslan C., Alagoz A.K. (2017). Building 3D anatomical model of coiling of the internal carotid artery derived from CT angiographic data. Eur. Arch. Oto-Rhino-Laryngol..

[10] Hussain R., Lalande A., Marroquin R., Guigou C., Grayeli A.B. (2020). Video-based augmented reality combining CT-scan and instrument position data to microscope view in middle ear surgery. Sci. Rep..

[11] Narayanan V., Narayanan P., Rajagopalan R., Karuppiah R., Rahman Z.A.A., Wormald P.-J., Van Hasselt C.A., Waran V. (2015). Endoscopic skull base training using 3D printed models with pre-existing pathology. Eur. Arch. Oto-Rhino-Laryngol..

[12] Cuello J.F., Saenz A., Liñares J.M., Martinez P., Ruiz C., Argañaraz R., Bailez M.M., Mantese B. (2020). Low-Cost Stereotactic Brain Biopsy Simulation Model. World Neurosurg..

[13] Chen R.K., Shih A.J. (2013). Multi-modality gellan gum-based tissue-mimicking phantom with targeted mechanical, electrical, and thermal properties. Phys. Med. Biol..

[14] Lai M., Skyrman S., Kor F., Homan R., Babic D., Edström E., Persson O., Burström G., Elmi-Terander A., Hendriks B.H.W. Development of a CT-compatible anthropomorphic skull phantom for surgical planning, training, and simulation. Proceedings of the 2021 SPIE Medical Imaging.

[15] Fedorov A., Beichel R., Kalpathy-Cramer J., Finet J., Fillion-Robin J.-C., Pujol S., Bauer C., Jennings D., Fennessy F., Sonka M. (2012). 3D Slicer as an image computing platform for the Quantitative Imaging Network. Magn. Reson. Imaging.

[16] Chan H.H.L., Siewerdsen J.H., Vescan A., Daly M.J., Prisman E., Irish J.C. (2015). 3D Rapid Prototyping for Otolaryngology—Head and Neck Surgery: Applications in Image-Guidance, Surgical Simulation and Patient-Specific Modeling. PLoS ONE.

[17] Weinstein M.A., Duchesneau P.M., MacIntyre W.J. (1977). White and Gray Matter of the Brain Differentiated by Computed Tomography. Radiology.

[18] Li Z., Ji C., Li D., Luo R., Wang G., Jiang J. (2020). A comprehensive study on the mechanical properties of different regions of 8-week-old pediatric porcine brain under tension, shear, and compression at various strain rates. J. Biomech..

[19] Forte A.E., Galvan S., Manieri F., Rodriguez y Baena F., Dini D. (2016). A composite hydrogel for brain tissue phantoms. Mater. Des..

[20] Skyrman S., Lai M., Edström E., Burström G., Förander P., Homan R., Kor F., Holthuizen R., Hendriks B.H.W., Persson O. (2021). Augmented reality navigation for cranial biopsy and external ventricular drain insertion. Neurosurg. Focus.

[21] Dho Y.-S., Lee D., Ha T., Ji S.Y., Kim K.M., Kang H., Kim M.-S., Kim J.W., Cho W.-S., Kim Y.H. (2021). Clinical application of patient-specific 3D printing brain tumor model production system for neurosurgery. Sci. Rep..

[22] Kunimatsu A., Kunimatsu N. (2017). Skull Base Tumors and Tumor-Like Lesions: A Pictorial Review. Pol. J. Radiol..

[23] Almefty K., Pravdenkova S., Colli B.O., Al-Mefty O., Gokden M. (2007). Chordoma and chondrosarcoma: Similar, but quite different, skull base tumors. Cancer.

[24] Bae J.W., Lee D.Y., Pang C.H., Kim J.E., Park C.-K., Lee D., Park S.J., Cho W.-S. (2021). Clinical application of 3D virtual and printed models for cerebrovascular diseases. Clin. Neurol. Neurosurg..

[25] Lai M., Shan C., Babic D., Homan R., Terander A.E., Edstrom E., Persson O., Burstrom G., de With P.H.N. Image fusion on the endoscopic view for endo-nasal skull-base surgery. Proceedings of the 2019 SPIE Medical Imaging.

[26] Lai M., Shan C., de With P.H.N. Hand-eye camera calibration with an optical tracking system. Proceedings of the 12th International Conference on Distributed Smart Cameras.

[27] Lai M., Skyrman S., Shan C., Babic D., Homan R., Edström E., Persson O., Burström G., Elmi-Terander A., Hendriks B.H.W. (2020). Fusion of augmented reality imaging with the endoscopic view for endonasal skull base surgery; a novel application for surgical navigation based on intraoperative cone beam computed tomography and optical tracking. PLoS ONE.

[28] Thiong’O G.M., Bernstein M., Drake J.M. (2021). 3D printing in neurosurgery education: A review. 3D Print. Med..

[29] Chawla S., Devi S., Calvachi P., Gormley W.B., Rueda-Esteban R. (2022). Evaluation of simulation models in neurosurgical training according to face, content, and construct validity: A systematic review. Acta Neurochir..

[30] Grillo F.W., Souza V.H., Matsuda R.H., Rondinoni C., Pavan T.Z., Baffa O., Machado H.R., Carneiro A.A.O. (2018). Patient-specific neurosurgical phantom: Assessment of visual quality, accuracy, and scaling effects. 3D Print. Med..

[31] Craven C., Baxter D., Cooke M., Carline L., Alberti S.J.M.M., Beard J., Murphy M. (2014). Development of a modelled anatomical replica for training young neurosurgeons. Br. J. Neurosurg..

[32] Waran V., Narayanan V., Karuppiah R., Owen S.L.F., Aziz T. (2014). Utility of multimaterial 3D printers in creating models with pathological entities to enhance the training experience of neurosurgeons: Technical note. J. Neurosurg..

[33] Saleh Y., Piper R., Richard M., Jeyaretna S., Cosker T. (2022). Designing a 3D Printed Model of the Skull-Base: A Collaboration Between Clinicians and Industry. J. Med. Educ. Curric. Dev..

[34] Guo X.-Y., He Z.-Q., Duan H., Lin F.-H., Zhang G.-H., Zhang X.-H., Chen Z.-H., Sai K., Jiang X.-B., Wang Z.-N. (2020). The utility of 3-dimensional-printed models for skull base meningioma surgery. Ann. Transl. Med..

[35] Levi D., Rampa F., Barbieri C., Pricca P., Franzini A., Pezzotta S. (2002). True 3D reconstruction for planning of surgery on malformed skulls. Child’s Nerv. Syst..

[36] Byvaltsev V., Polkin R., Bereznyak D., Giers M.B., Hernandez P.A., Shepelev V., Aliyev M. (2021). 3D-printed cranial models simulating operative field depth for microvascular training in neurosurgery. Surg. Neurol. Int..

[37] Mashiko T., Otani K., Kawano R., Konno T., Kaneko N., Ito Y., Watanabe E. (2015). Development of Three-Dimensional Hollow Elastic Model for Cerebral Aneurysm Clipping Simulation Enabling Rapid and Low Cost Prototyping. World Neurosurg..

[38] Liu Y., Gao Q., Du S., Chen Z., Fu J., Chen B., Liu Z., He Y. (2017). Fabrication of cerebral aneurysm simulator with a desktop 3D printer. Sci. Rep..

[39] Iida H., Hori Y., Ishida K., Imabayashi E., Matsuda H., Takahashi M., Maruno H., Yamamoto A., Koshino K., Enmi J. (2012). Three-dimensional brain phantom containing bone and grey matter structures with a realistic head contour. Ann. Nucl. Med..

[40] Zhuang Y.-D., Zhou M.-C., Liu S.-C., Wu J.-F., Wang R., Chen C.-M. (2019). Effectiveness of personalized 3D printed models for patient education in degenerative lumbar disease. Patient Educ. Couns..

[41] van de Belt T.H., Nijmeijer H., Grim D., Engelen L.J.L.P.G., Vreeken R., van Gelder M.M.H.J., ter Laan M. (2018). Patient-Specific Actual-Size Three-Dimensional Printed Models for Patient Education in Glioma Treatment: First Experiences. World Neurosurg..

[42] Akkus Z., Galimzianova A., Hoogi A., Rubin D.L., Erickson B.J. (2017). Deep Learning for Brain MRI Segmentation: State of the Art and Future Directions. J. Digit. Imaging.

